# Systemic metabolic engineering of *Enterobacter aerogenes* for efficient 2,3-butanediol production

**DOI:** 10.1007/s00253-023-12911-8

**Published:** 2024-01-19

**Authors:** Ping Lu, Ruoxuan Bai, Ting Gao, Jiale Chen, Ke Jiang, Yalun Zhu, Ye Lu, Shuting Zhang, Fangxu Xu, Hongxin Zhao

**Affiliations:** 1 Zhejiang Province Key Laboratory of Plant Secondary Metabolism and Regulation, College of Life Sciences and Medicine, Zhejiang Sci-Tech University, Hangzhou, 310018 China; 2 Liaoning Province Key Laboratory of Cordyceps Militaris With Functional Value, Experimental Teaching Center, Shenyang Normal University, Shenyang, 110034 China

**Keywords:** DR1558, *Enterobacter aerogenes*, Systemic metabolic engineering, Tolerance, 2,3-Butanediol

## Abstract

**Abstract:**

2,3-Butanediol (2,3-BDO) is an important gateway molecule for many chemical derivatives. Currently, microbial production is gradually being recognized as a green and sustainable alternative to petrochemical synthesis, but the titer, yield, and productivity of microbial 2,3-BDO remain suboptimal. Here, we used systemic metabolic engineering strategies to debottleneck the 2,3-BDO production in *Enterobacter aerogenes*. Firstly, the pyruvate metabolic network was reconstructed by deleting genes for by-product synthesis to improve the flux toward 2,3-BDO synthesis, which resulted in a 90% increase of the product titer. Secondly, the 2,3-BDO productivity of the IAM1183-LPCT/D was increased by 55% due to the heterologous expression of DR1558 which boosted cell resistance to abiotic stress. Thirdly, carbon sources were optimized to further improve the yield of target products. The IAM1183-LPCT/D showed the highest titer of 2,3-BDO from sucrose, 20% higher than that from glucose, and the yield of 2,3-BDO reached 0.49 g/g. Finally, the titer of 2,3-BDO of IAM1183-LPCT/D in a 5-L fermenter reached 22.93 g/L, 85% higher than the wild-type strain, and the titer of by-products except ethanol was very low.

**Key points:**

- *Deletion of five key genes in E. aerogenes improved 2,3-BDO production*
- *The titer of 2,3-BDO was increased by 90% by regulating metabolic flux*
- *Response regulator DR1558 was expressed to increase 2,3-BDO productivity*

**Graphical abstract:**

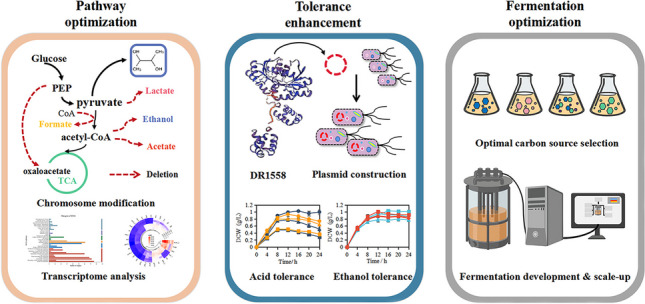

**Supplementary Information:**

The online version contains supplementary material available at 10.1007/s00253-023-12911-8.

## Introduction

Growing environmental pollution and energy security concerns are drawing more attention to renewable energy and sustainable chemical manufacturing (Liang et al. 2020). 2,3-Butanediol (2,3-BDO) is a promising bulky platform biochemical (Song et al. 2019), which is a gateway molecule for many chemical derivatives. For example, it is applied to produce 1,3-butadiene, which can then be changed into nylon and synthetic resins (Boecker et al. 2021; Liu et al. 2016). 2,3-BDO, which has a low freezing point of − 60 °C, is utilized as antifreeze (Tinôco et al. 2021; Celińska and Grajek 2009). Besides, 2,3-BDO can be dehydrated to produce methyl ethyl ketone (Lee and Seo 2019), a fuel additive with superior combustion heat than ethanol (29,005 J/g) that is also used as a solvent for resins and lacquers (Hakizimana et al. 2019). 2,3-BDO is currently roughly $3.23/kg, and by 2030, the global market is anticipated to achieve $17.71 billion (Narisetty et al. 2022). However, while its market demand is clearly growing, the production of 2,3-BDO via the non-specific hydrolysis of petroleum-derived four-carbon hydrocarbons is also a highly energy-intensive process with high production costs and environmental pollution (Chu et al. 2021). Therefore, microbial production is gradually being recognized as a cost-effective and environmentally friendly alternative for 2,3-BDO production. However, the existing bioproduction methods of 2,3-BDO are still far from industrial production and commercial utilization in terms of production stability and production scale. Therefore, an in-depth study on the existing fermentation method for the biological production of 2,3-BDO to explore a more stable performance of 2,3-BDO production and to realize the industrialization of aerobic fermentation for the biological production of 2,3-BDO at an early date has become the focus of current research on the fermentation method for the production of 2,3-BDO.

*Enterobacter aerogenes* is a facultatively anaerobic Gram-negative bacterium (Wu et al. 2021), which is one of the most popular strains in the study of green bioenergy in recent years. *E. aerogenes* can naturally produce 2,3-BDO with a broad substrate spectrum and short growth cycle and is appropriate for high-cell-density fermentation (Lu et al. 2022). In addition, *E. aerogenes* also has the advantages of clear physiological and biochemical characteristics, clear genetic background, and mature molecular manipulation and metabolic regulation techniques. Therefore, *E. aerogenes* is an ideal strain for the green production of 2,3-BDO using renewable resources, as well as an ideal platform for creating high-performance industrial strains.

Optimization of metabolic pathways is an important strategy for improving strain productivity. Like many other bacteria, *E. aerogenes* exhibits mixed acid fermentation, producing a large number of by-products as part of spillover metabolism (Maina et al. 2022). The limited supply of precursors is one of the main obstacles in the synthesis of target products by microbial hosts. Most of the by-products produced by *E. aerogenes* mixed acid fermentation compete with 2,3-BDO for the precursors and cofactors. Therefore, in order to effectively increase the titer of 2,3-BDO, comprehensive diagnosis and optimization of the strain’s metabolic state is crucial research content. This involves not only removing the metabolic flux bottleneck but also transferring as much carbon flux and cofactors as possible to the formation of the final product (Lee and Kim 2015). Previous studies used genetic engineering to restrict the generation of by-products during the synthesis of 2,3-BDO. Ge et al. engineered *Klebsiella pneumoniae* by deleting *ldhA* and *ack*, which reduced the formation of by-products, while enhancing the 2,3-BDO biosynthesis (Ge et al. 2020). When Thapa et al. modified *E. aerogenes* by eliminating *ldh*A and *pta*, the 2,3-BDO titer in flask cultivation increased 8.11 times above the wild type (Thapa et al. 2019). Thus, increasing the biosynthesis of 2,3-BDO through restricting pathways that compete for NADH and carbon is a possible option (Wu et al. 2021).

The intrinsic toxicity of compounds such as alcohols and carboxylic acids at high concentrations leads to the inhibition of cell development and metabolism, which is one of the factors that hinder microbial production of target products at high levels (Nicolaou et al. 2010). Carboxylic acid and alcohol metabolites produced by mixed acid fermentation of *E. aerogenes* are known to inhibit cell growth, thereby affecting the accumulation of high levels of 2,3-BDO. Currently, engineering strategies that increase cellular stress tolerance have been widely used to improve microbial productivity (Appukuttan et al. 2015). *Deinococcus radiodurans* is one of the most stress-tolerant species discovered to date (Krisko and Radman 2013), and its two-component response regulator DR1558 has been demonstrated to trigger cell alterations in response to environmental stress by directly binding to gene promoter regions or attaching to effector molecules (Park et al. 2020a, b). In recent studies, DR1558 has been used to improve the tolerance of *E. coli* to low pH, alcohol, and salt stress (Park et al. 2020a, b). In addition, DR1558 has been applied to increase cellular resilience and the generation of succinate (Guo et al. 2017), GABA (Park et al. 2020a, b), and poly-3-hydroxybutyrate (PHB) (Park et al. 2019) in recombinant *E. coli* strains.

**Fig. 1:**
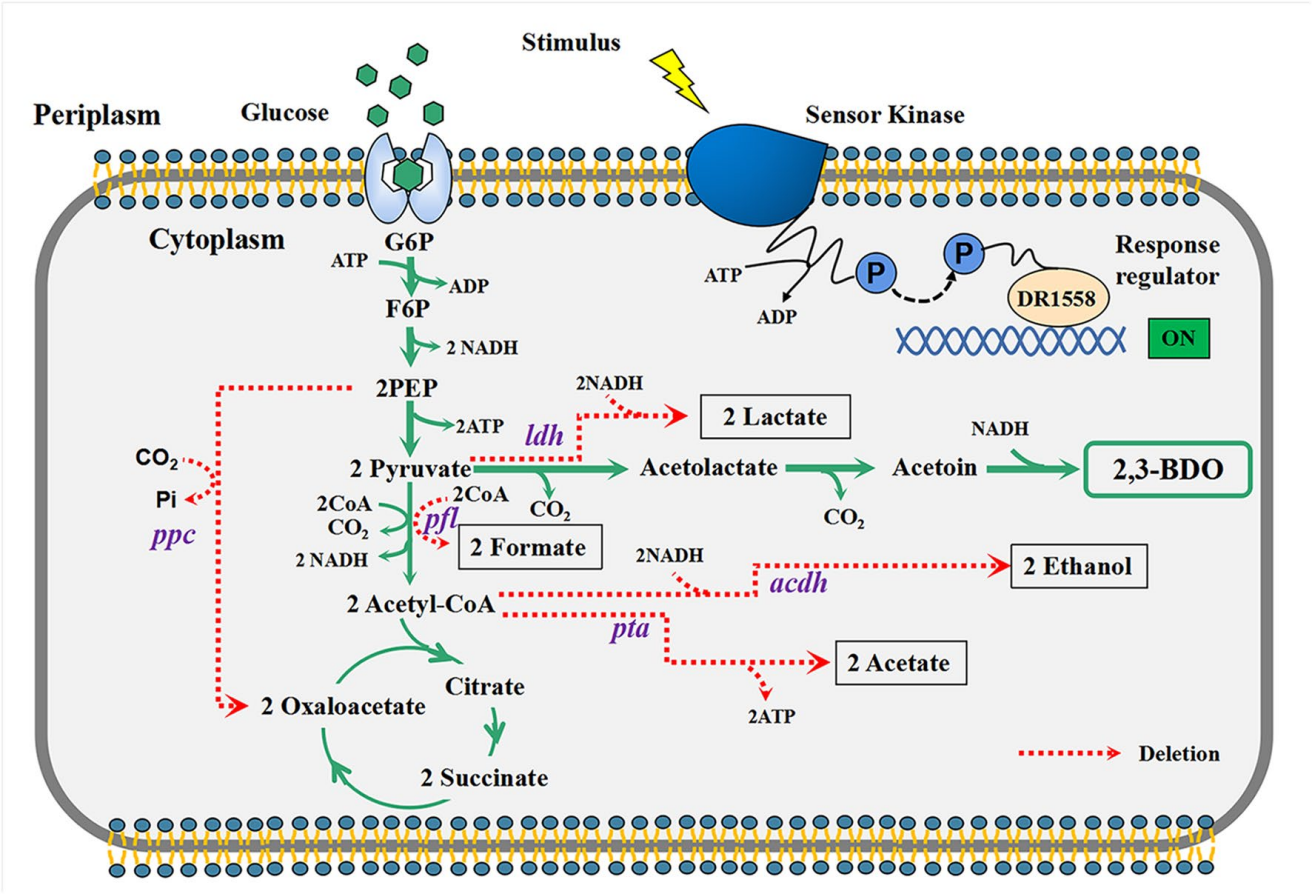
Synthesis of 2,3-butanediol metabolic route and metabolic engineering objectives. Genes: *ldh*, lactate dehydrogenase; *pfl*, pyruvate formate-lyase; *ppc*, phosphoenolpyruvate carboxylase; *pta*, phosphate acetyltransferase; *acdh*, acetaldehyde dehydrogenase

Systemic metabolic engineering (SME), which combines the methods and techniques of systems biology, synthetic biology, and evolutionary engineering with classical metabolic engineering, has been adopted in recent years to promote the creation of high-performance strains (Lee and Kim 2015; Choi et al. 2019). Based on the research progress of systemic metabolic engineering and the in-depth understanding of the whole gene expression of strains through transcriptomic analysis, a more comprehensive and systematic study of 2,3-BDO synthetic pathways was carried out to reasonably design and develop an engineered strain with industrial application potential. In this work, *E. aerogenes* IAM1183 was selected as the starting strain and the production of 2,3-BDO was increased by multi-strategy engineering. First, we evaluated the effects of blocking different by-product pathways on host strain growth and 2,3-BDO production. Then, the pyruvate metabolic network was reconstructed to promote the flux toward the 2,3-BDO synthesis. Transcriptome analysis was used to further study the specific metabolic status of cells. In addition, we introduced the response regulator DR1558 into the host strain to increase overall cellular tolerance, thereby further increasing the productivity of 2,3-BDO (Fig. 1). This study provides a useful combinatorial approach for improving industry-relevant microbial production.

## Materials and methods

### Strains, plasmids, and reagents

All strains, plasmids, and primers used are shown in Tables 1 and 2. Primer synthesis and genome sequencing were commissioned to Sangon (Shanghai, China). The DNA polymerases used such as the Phanta® Max Super-Fidelity DNA Polymerase and 2 × Taq Master Mix (Dye Plus) were purchased from Vazyme (Nanjing, China). All restriction enzymes (*Sac* I, *Xba* I, *Eco*R I, *Hin*d III) were obtained from Takara Bio. Inc. (Beijing, China).

**Table 1:** Strains and plasmids used in this study

Strains and plasmids	Genotype or relevant characteristics	Source or reference
Strains		
IAM1183	Wild type, *E. aerogenes*	Wu et al. (2021)
IAM1183-L	IAM1183 Δ*ldh*	Lu et al. (2022)
IAM1183-P	IAM1183 Δ*pfl*	Lu et al. (2022)
IAM1183-C	IAM1183 Δ*ppc*	This study
IAM1183-T	IAM1183 Δ*pta*	This study
IAM1183-H	IAM1183 Δ*acdh*	This study
IAM1183-LP	IAM1183 Δ*ldh* Δ*pfl*	Lu et al. (2022)
IAM1183-LPC	IAM1183 Δ*ldh* Δ*pfl* Δ*ppc*	This study
IAM1183-LPCT	IAM1183 Δ*ldh* Δ*pfl* Δ*ppc* Δ*pta*	This study
IAM1183-LPCH	IAM1183 Δ*ldh* Δ*pfl* Δ*ppc* Δ*acdh*	This study
IAM1183/D	IAM1183, carrying plasmid pET-28a-1558	This study
IAM1183-LP/D	IAM1183 Δ*ldh* Δ*pfl*, carrying plasmid pET-28a-1558	This study
IAM1183-LPC/D	IAM1183 Δ*ldh* Δ*pfl* Δ*ppc*, carrying plasmid pET-28a-1558	This study
IAM1183-LPCT/D	IAM1183 Δ*ldh* Δ*pfl* Δ*ppc* Δpta, carrying plasmid pET-28a-1558	This study
IAM1183-LPCH/D	IAM1183 Δ*ldh* Δ*pfl* Δ*ppc* Δ*acdh*, carrying plasmid pET-28a-1558	This study
*E. coil* DH5α	F^–^Φ80d/*lac*ZΔM15, Δ(*lac*ZYA-*arg*F) U169, *recA*1, *endA*1, *hsdR*17 (r_K_^–^, m_K_^+^), *phoA*, *supE*44, λ-, *thi*-1, *gyrA*96, *relA*1	Vazyme
*E. coil* BL21 (DE3)	F^–^, *omp*T, *hsd*S_B_ (r_B_^−^ m_B_^−^), *gal*, *dcm* (DE3)	Vazyme
*E. coil* S17-1 λpir	*rec*A *pro hsd*R RP4-2-Tc::Mu-Km::Tn7; mobilizer strain	Li et al. (2018)
HB101/pRK2013	HB101 harboring pRK2013; Km^r^	Li et al. (2018)
*Deinococcus radiodurans*	Wild type	Yang et al. (2014)
Plasmid		
pRK2013	ColE1 *mob* + *tra*RK2Δ*rep*RK2*rep*E^−^ Km^r^	Li et al. (2018)
pLO3	Tc^r^ *sacB*, RP4 *oriT*, ColE1 *ori*	Li et al. (2018)
pLO3-ppc	pLO3 carrying upstream 664 bp and downstream 578 bp of *ppc*	This study
pLO3-pta	pLO3 carrying upstream 450 bp and downstream 583 bp of *pta*	This study
pLO3-acdh	pLO3 carrying upstream 643 bp and downstream 494 bp of *acdh*	This study
pET-28a ( +)	Expression vector, Km^r^	Takara
pET-28a-1558	pET-28a derivative containing *DR1558*, Km^r^	This study

**Table 2:** Primers used in this study

Primers	Sequence (5′ to 3′)	Source
ppc-UF	TAGGAGCTCACGCCTCAAACCGCATCTGC (*Sac* I)	This study
ppc-UR	TACCGGGAACGGCTGATAAAGTTACCCCAGACACCCCATC	This study
ppc-DF	GATGGGGTGTCTGGGGTAACTTTATCAGCCGTTCCCGGTA	This study
ppc-DR	TGCTCTAGAGGATCTGGCAGTCGGTGA (*Xba* I)	This study
pta-UF	TAGGAGCTCCGTGCAATGGACGTTTACTG (*Sac* I)	This study
pta-UR	CAGGCGGATAATCCAGCAGACAGCTTCCCGTTATATTTCA	This study
pta-DF	TGAAATATAACGGGAAGCTGTCTGCTGGATTATCCGCCTG	This study
pta-DR	TGCTCTAGACGATGCGACGATGTTTGAGG (*Xba* I)	This study
acdh-UF	TAGGAGCTCGGAGAACGCAAATGGGTGA (*Sac* I)	This study
acdh-UR	TATCGCCTTATTTCAATGCGGAGTCTATCCTTCATCAGAC	This study
acdh-DF	GTCTGATGAAGGATAGACTCCGCATTGAAATAAGGCGATA	This study
acdh-DR	TGCTCTAGATCCGCTACCACGTCATCCCA (*Xba* I)	This study
DR1558-F	CCGGAATTCGTGACTCTGCCTCAAGGAG (*Eco*R I)	This study
DR1558-R	CAAGCTTTTCACAACTCCACGCCCTCC (*Hin*d III)	This study

Relevant restriction enzyme sites are underlined

### Medium and culture conditions

*D. radiodurans* was cultivated in TGY media (10 g/L tryptone, 3 g/L yeast extract, and 1 g/L glucose). Strains for cloning and genetic manipulation were grown in Luria–Bertani broth at 30 °C or 37 °C as required with 220 rpm shaking. For selection or induction, streptomycin sulfate, tetracycline, kanamycin, ampicillin, and isopropyl-β-D-thiogalactopyranoside (IPTG) were added, when necessary. For evaluating 2,3-BDO production in fermentation, all strains were cultivated in aerobic fermentation medium containing 1.2 mg/L ZnCl_2_, 1.2 mg/L FeCl_3_·6H_2_O, 1.2 mg/L MnCl_2_·4H_2_O, 1.2 mg/L CuCl_2_·2H_2_O, 3.1 mg/L H_3_BO_3_, 0.26 g/L MgSO_4_·7H_2_O, 0.28 g/L Na_2_SO_4_, 0.42 g/L citric acid, 0.75 g/L KCl, 3 g/L KH_2_PO_4_, 5 g/L yeast extract, 5.35 g/L (NH_4_)_2_SO_4_, 6.8 g/L Na_2_HPO_4_, and 30 g/L sugar (Jung et al. 2014).

For the shake-flask fermentations, single colonies were grown in 50 mL of Luria–Bertani broth at 37 °C for 12 h while being shaken at 220 rpm to provide seed cultures. After that, the seed cultures were put into fermentation bottles with a capacity of 250 mL and 50 mL of aerobic fermentation medium, and they fermented for 24 h. To maintain the consistency of biological inoculation amount of parallel samples, the inoculation volume was determined using the following formula: *V* = 50/(51*X − *1), where *V* was the inoculum volume and *X* was the OD_600_ of the strain.

For the batch fermentation, 50 mL of OD_600_ = 2 seed culture was inoculated with 3-L aerobic fermentation medium, 60 g/L sucrose was added, and aerobic incubation was carried out at 37 °C and 150 rpm for 60 h. Sterile air was charged at a flow rate of 3 L/min. At intervals of every 2 h, samples of the fermentation liquid were collected.

### Plasmid construction

The coding sequence of DR1558 was amplified from the genome of *D. radiodurans* using the primers DR1558-F and DR1558-R, digested with *Eco*R I/*Hin*d III, and ligated into the pET-28a vector, yielding the recombinant plasmid pET-28a-1558, which was further confirmed by sequencing. The pET-28a-1558 was then electroporated into both the IAM1183 and the gene combination knockout mutant. By adding 0.1 mM (final concentration) IPTG for 6 h, the heterologous expression of DR1558 was induced, and this was then verified by SDS-PAGE (Fig. S1).

### Strain construction

The *ppc* gene deletion mutant was created using the suicide vector pLO3 made available by Li et al. (Li et al. 2018). By utilizing the primers ppc-UF/UR and ppc-DF/UR, respectively, and an overlap-extension PCR reaction, the upstream and downstream homologous arms of the *ppc* gene were amplified from the deoxyribonucleic acid of IAM1183 and fused. To create plasmid pLO3-ppc, the homologous segment was ligated into the pLO3 vector, which had already been digested with the same enzymes (*Sac* I/*Xba* I). *E. coli* S17-1 was given the plasmid pLO3-ppc, creating strain *E. coli* S17-1/pLO3-ppc.

With *E. coli* HB101/pRK2013 as an auxiliary strain, the pLO3-ppc suicide vector was transmitted from *E coli* S17-1/pLO3-ppc into *E. aerogenes* through triparental filter mating (Figurski and Helinski 1979; Harding et al. 2006). The *ppc* gene deletion mutants were isolated by selecting for lack of sucrose (8%) sensitivity (Li et al. 2018), and the correctly deleted clone was tested by PCR. To examine the genetic stability of the mutants, the strains were serially inoculated into fresh Luria–Bertani broth and cultured continuously for 3 days. Sequencing was used to confirm the *ppc*-deficient IAM1183 mutant, designated as IAM1183-C. All other mutants were constructed in the same manner.

### Transcriptome sequencing (RNA-Seq)

Cells were collected from shaker culture during the exponential growth phase (6 h). RNA was isolated by utilizing the TRIzol procedure (Rio et al. 2010). Following the RNAtag-Seq protocol, RNA oligo linkers were used for labeling, pooling, ribosome depletion, and library construction (Shishkin et al. 2015). The final sample was sequenced on Illumina NovaSeq 6000. Finally, the sequencing data were processed and analyzed using Rockhopper. All sequence data has been deposited to the SRA database (PRJNA998181).

### Real-time quantitative PCR (qPCR) analysis

The TRIzol technique was used to collect cellular total RNA, which was then reverse transcribed into cDNA using the ReverTra® Ace qPCR RT Master Mix with gDNA Remover Kit (TOYOBO, Japan). qPCR was carried out using a SYBR® Green Realtime PCR Master Mix Kit (TOYOBO, Japan) on a real-time fluorescence quantitative PCR apparatus (ABI 7500, Meixuan, China). The qPCR procedure involves a 60-s initial denaturation at 95 °C, followed by 40 cycles of 15 s at 95 °C and 40 s at 60 °C. With *RecA* acting as the internal reference gene (Gomes et al. 2018), the 2^−△△CT^ approach was utilized to quantify the transcription levels of the relevant genes and normalize them to IAM1183.

### Analytical methods

To determine the concentration of metabolites, 2 mL of culture supernatants that had been centrifuged at 10,000 rpm for 10 min was filtered through a 0.2-mm syringe filter before being pipetted into the chromatographic sample tube. The high-performance liquid chromatography (HPLC) (LC-20A, Shimadzu, Japan) was used to determine the concentrations of formate, lactate, acetate, pyruvate, acetoin, and succinate using a Shimadzu PREP-ODS(H) column and a Shimadzu RID-10A refractive index detector with 0.2% aqueous phosphoric acid at a flow rate of 0.8 mL/min. The GC system (GC-2010, Shimadzu, Japan) was used to determine the concentrations of alcohols using a Parapak Q column with He at a flow rate of 1 mL/min. The concentrations of analytes were calculated by contrasting the peak sizes to a standard curve constructed for each compound.

## Results

### Engineering of IAM1183 for 2,3-BDO production

*E. aerogene*s IAM1183 was genetically modified to determine the effects of blocking different by-product biosynthesis pathways on cell growth and 2,3-BDO formation. Attempts were made to delete the genes encoding lactate dehydrogenase (*ldh*), pyruvate formate lyase (*pfl*), phosphoenolpyruvate carboxylase (*ppc*), phosphate acetyltransferase (*pta*), and acetaldehyde dehydrogenase (*acdh*) in wild type to disrupt the production of carboxylic acids and alcohols, the main by-products of 2,3-BDO synthesis (Fig. 1). The IAM1183-C (*Δppc*), IAM1183-T (*Δpta*), and IAM1183-H (*Δacdh*) were successfully constructed in this study, and the IAM1183-L (*Δldh*) and IAM1183-P (*Δpfl*) have been successfully obtained in our previous research (Lu et al. 2022).

**Fig. 2:**
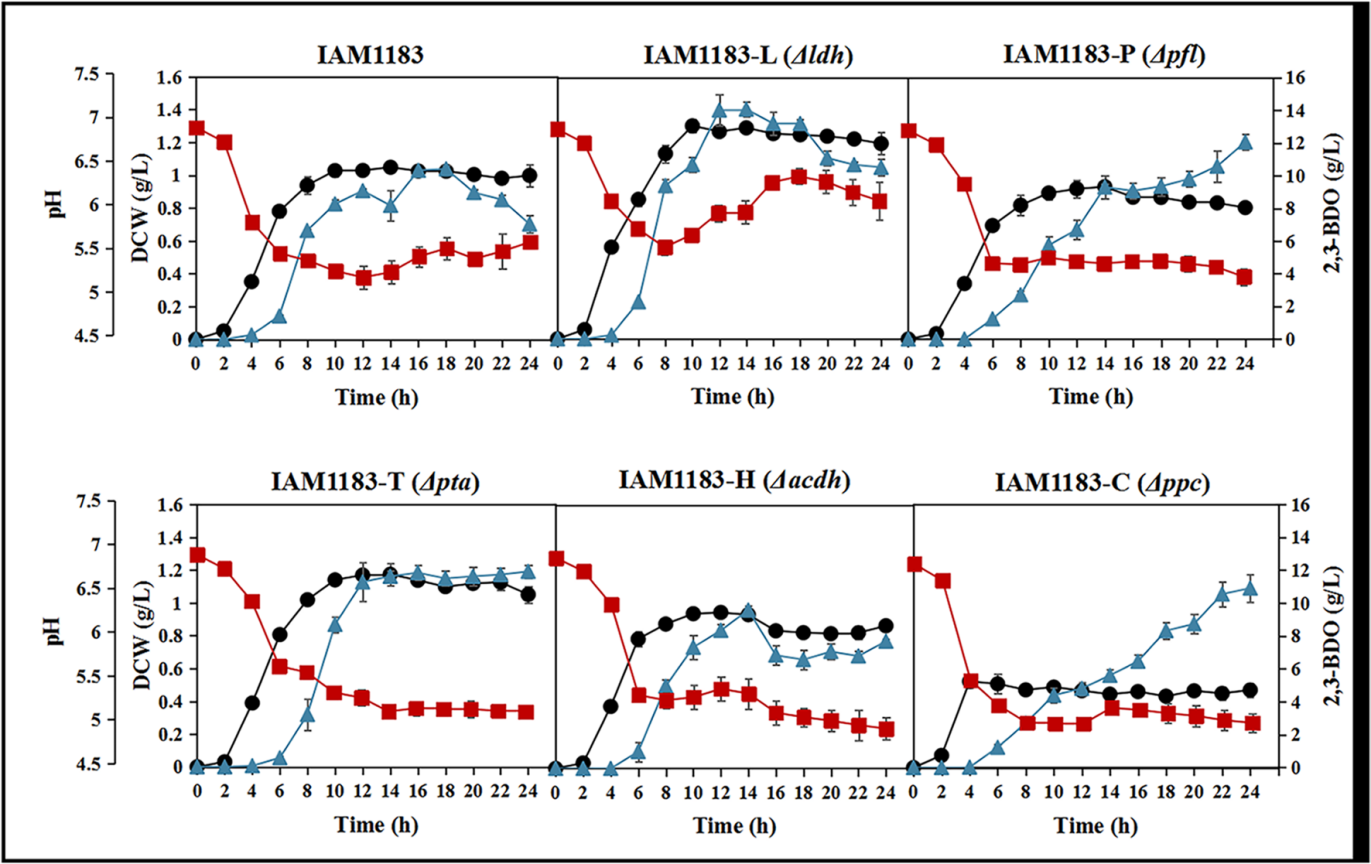
Effects of different gene knockout on cell growth and 2,3-butanediol production. Symbols: DCW (black-filled circle), 2,3-BDO (blue-filled triangle), and pH (red-filled square). DCW means dry cell weight. *n* = 3 for each curve. Error bars show mean value ± SD

All strains produced 2,3-BDO with an initial glucose concentration of 30 g/L under aerobic conditions. As anticipated, the deletion of the *ldh* and *pta* genes significantly increased the cell growth (Fig. 2). However, the deletion of the *pfl*, *ppc*, and *acdh* genes reduced growth at varying degrees compared to the original strain IAM1183, with the *acdh* gene deletion showing the smallest decrease and the *ppc* gene deletion the largest (Fig. 2). The strain IAM1183-C with the *ppc* gene deletion reached the stationary phase after 4 h, while the other mutants took 10 h before entering the growth plateau.

In terms of 2,3-BDO production, the highest 2,3-BDO production achieved by all mutant strains was higher than that of the original strain IAM1183, with IAM1183-L being the highest (Fig. 2). However, it is worth noting that strains IAM1183-P and IAM1183-C showed a different trend of product accumulation from other strains (Fig. 2). While the metabolic concentration trend of 2,3-BDO of other strains was slowly accumulated or even gradually depleted as the growth trend entered a plateau, the 2,3-BDO production of IAM1183-P and IAM1183-C mutant strains was continuously accumulated during 24-h fermentation culture.

After 24-h aerobic fermentation, the titer of 2,3-BDO produced by all the mutant strains significantly increased compared to the original strain, with strain IAM1183-P showing the highest titer, followed by IAM1183-T, IAM1183-C, IAM1183-L, and IAM1183-H (Table 3). In addition, all mutant strains had lower concentrations of most by-products than the original strain IAM1183 (Table 3).

**Table 3:** Flask cultivation with host strain and single knockout engineered strain

Strains	Lactate (g/L)	Formate (g/L)	Acetate (g/L)	Succinate (g/L)	Pyruvate (g/L)	Ethanol (g/L)	Acetoin (g/L)	2,3-BDO (g/L)	Glucose (g/L)
IAM1183-L	0.11 ± 0.01	0.54 ± 0.03	0.62 ± 0.01	0.52 ± 0.03	0.10 ± 0.01	0.52 ± 0.02	2.51 ± 0.08	10.52 ± 0.50	3.65 ± 0.18
IAM1183-P	1.39 ± 0.03	0.57 ± 0.05	0.88 ± 0.02	0.35 ± 0.07	0.19 ± 0.01	0.50 ± 0.03	1.84 ± 0.10	12.06 ± 0.46	2.14 ± 0.28
IAM1183-C	2.80 ± 0.26	1.41 ± 0.02	0.62 ± 0.05	0.21 ± 0.02	0.22 ± 0.01	0.44 ± 0.01	1.47 ± 0.10	10.90 ± 0.84	7.25 ± 0.83
IAM1183-T	2.07 ± 0.22	1.13 ± 0.04	0.34 ± 0.01	1.33 ± 0.12	0.19 ± 0.01	1.53 ± 0.13	2.07 ± 0.09	11.91 ± 0.40	1.88 ± 0.13
IAM1183-H	1.73 ± 0.13	1.43 ± 0.13	1.44 ± 0.05	1.16 ± 0.09	0.18 ± 0.01	0.38 ± 0.01	1.14 ± 0.08	7.72 ± 0.14	5.69 ± 0.44
IAM1183 (wild type)	1.87 ± 0.03	1.52 ± 0.04	1.38 ± 0.02	0.42 ± 0.06	0.18 ± 0.01	1.19 ± 0.11	2.80 ± 0.17	7.02 ± 0.51	3.67 ± 0.13

### Increasing flux through the 2,3-BDO biosynthesis pathway by reconstructing the pyruvate metabolic network

In order to construct the optimal mutant with a higher production of 2,3-BDO, five key genes related to the synthesis of by-products (*ldh*, *pfl*, *ppc*, *pta*, and *acdh*) were sequentially knocked out, resulting in the engineered strains IAM1183-LP (*Δldh Δpfl*), IAM1183-LPC (*Δldh Δpfl Δppc*), IAM1183-LPCT (*Δldh Δpfl Δppc Δpta*), and IAM1183-LPCH (*Δldh Δpfl Δppc Δacdh*). However, construction of the quintuple knockout mutant (*Δldh Δpfl Δppc Δpta Δacdh*) was unsuccessful for unknown reasons.

**Fig. 3:**
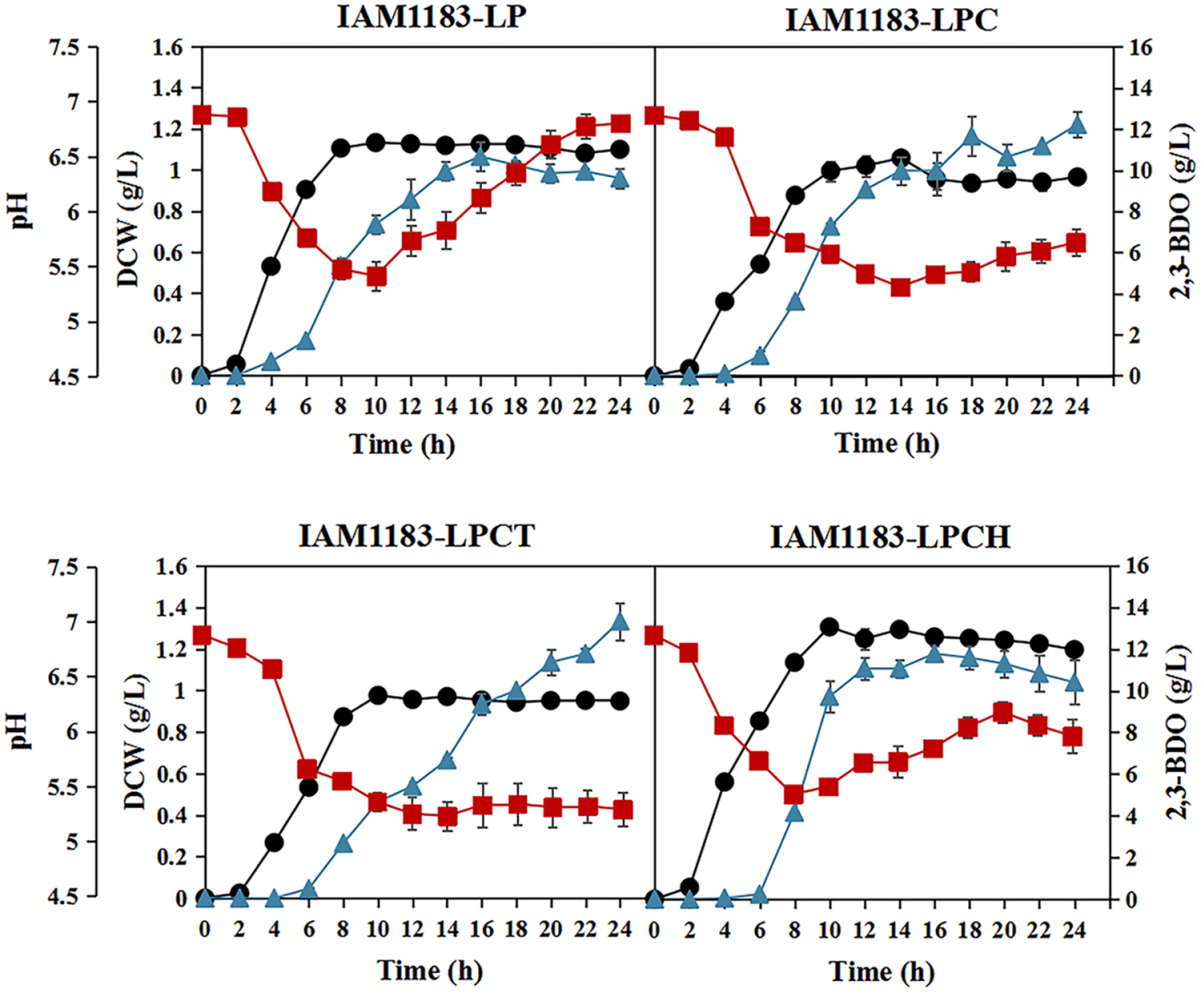
Effects of different gene combinations knockout on cell growth and 2,3-butanediol production. Symbols: DCW (black-filled circle), 2,3-BDO (blue-filled triangle), and pH (red-filled square). DCW means dry cell weight. *n* = 3 for each curve. Error bars show mean value ± SD

According to the biomass changes of the mutants within 24 h, multiple gene knockout had no detrimental effect on the growth and instead promoted the growth of the IAM1183-LP and IAM1183-LPCH. And the pH of the medium was also upregulated in both strains (Fig. 3). Meanwhile, the titer of 2,3-BDO of both IAM1183-LP and IAM1183-LPCH peaked at 16 h, and the titer in the later stage decreased slowly with the extension of fermentation time (Fig. 3). However, the IAM1183-LPC and IAM1183-LPCT showed different trends of 2,3-BDO accumulation, and both showed increasing trends of 2,3-BDO accumulation with increasing fermentation time. The IAM1183-LPCT produced 2,3-BDO at the highest titer (13.33 g/L), 90% greater than the wild type (7.02 g/L). Meanwhile, the yield of IAM1183-LPCT was also the highest (0.48 g/g), 78% higher than the wild type (0.27 g/g). The IAM1183-LP, IAM1183-LPC, and IAM1183-LPCH strains produced 2,3-BDO at 0.34, 0.43, and 0.43 g/g, respectively (Table 4).

**Table 4:** Flask cultivation with combined knockout engineered strain

Strains	Lactate (g/L)	Formate (g/L)	Acetate (g/L)	Succinate (g/L)	Pyruvate (g/L)	Ethanol (g/L)	Acetoin (g/L)	2,3-BDO (g/L)	Glucose (g/L)
IAM1183-LP	0.21 ± 0.02	0.25 ± 0.03	0.53 ± 0.02	0.58 ± 0.06	0.16 ± 0.02	0.86 ± 0.05	3.36 ± 0.08	9.59 ± 0.38	2.48 ± 0.19
IAM1183-LPC	0.97 ± 0.05	0.82 ± 0.03	0.88 ± 0.03	0.36 ± 0.05	0.20 ± 0.01	0.77 ± 0.02	1.80 ± 0.17	12.22 ± 0.53	2.09 ± 0.11
IAM1183-LPCT	1.13 ± 0.12	0.71 ± 0.02	0.35 ± 0.10	0.27 ± 0.10	0.19 ± 0.01	1.26 ± 0.08	2.24 ± 0.24	13.33 ± 1.09	2.27 ± 0.14
IAM1183-LPCH	1.01 ± 0.09	0.65 ± 0.04	0.92 ± 0.7	0.37 ± 0.07	0.20 ± 0.01	0.61 ± 0.03	2.87 ± 0.02	11.75 ± 0.72	2.99 ± 0.26

**Fig. 4:**
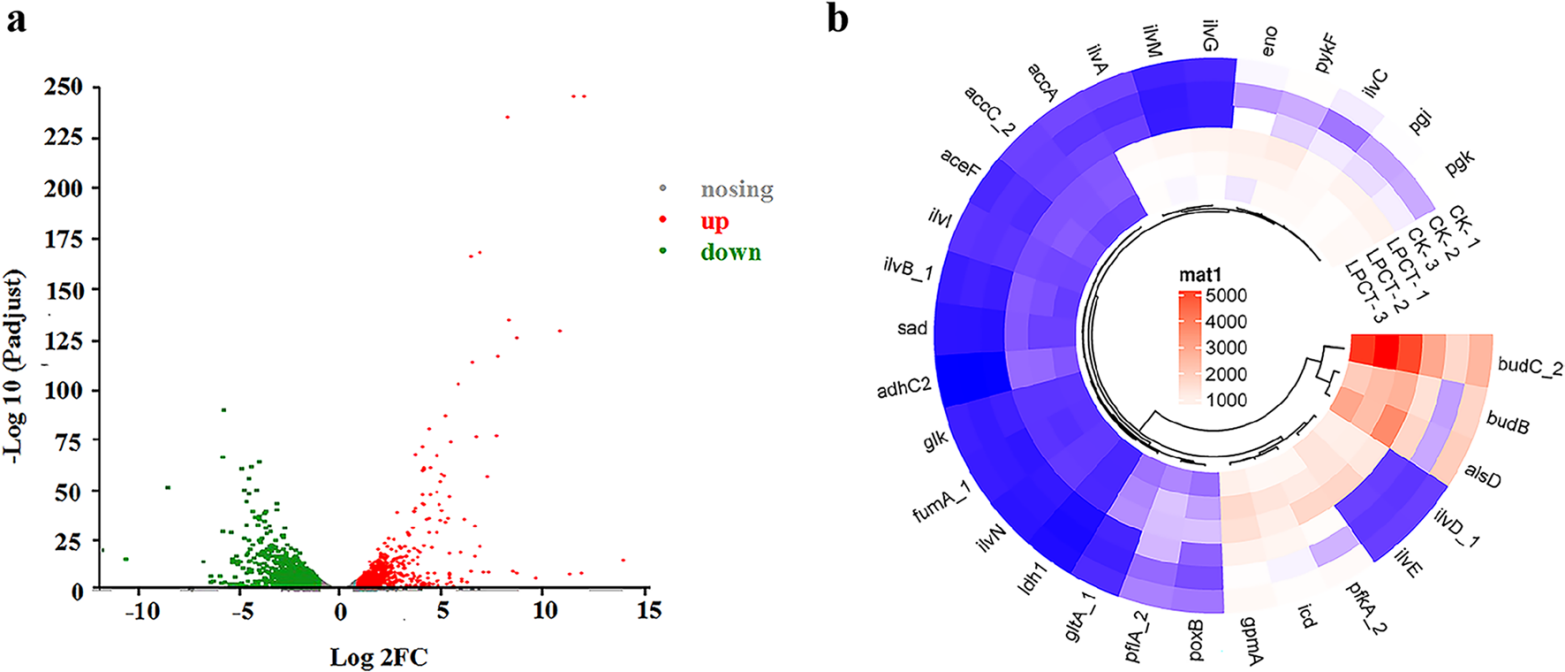
Genome-wide transcriptional analysis of IAM1183-LPCT by RNA-Seq. **a** Profiles of gene transcription differences. **b** Transcriptional upregulation amplitude of upregulated genes related to 2,3-BDO synthesis pathway. CK-1, 2, 3 are three replicates of strain IAM1183; LPCT-1, 2, 3 are three replicates of strain IAM1183-LPCT

In order to analyze the change of the gene expression level of engineering strain IAM1183-LPCT during the 2,3-BDO biosynthesis, RNA-Seq technology was used to analyze the transcriptome of the IAM1183-LPCT in the exponential growth stage. By using the genomic information of the original strain IAM1183 as a control, the results of the engineered strain IAM1183-LPCT showed that 1822 genes were significantly expressed, and the differential performance of the upregulated genes was more significant than the downregulated genes (Fig. 4a). In order to better understand the effect of gene modification on 2,3-BDO production by the IAM1183-LPCT, the genes that were significantly upregulated in the pyruvate metabolic pathway were explored and summarized in Fig. 4a (see Table S1 for gene name annotation). After gene deletions, the expression of genes encoding three key enzymes in 2,3-BDO synthesis, diacyl reductase (*budC*_2), α-acetolactate decarboxylase (*alsD*), and α-acetolactate synthase (*budB*), was significantly upregulated in the IAM1183-LPCT, with *budC*_2 being the most significantly upregulated, with nearly threefold upregulation compared to the original strain IAM1183 (Fig. 4b). In addition, several acetolactate synthase isozymes that catalyze the conversion of pyruvate to acetolactate were found significantly upregulated in IAM1183-LPCT after the gene deletion (Fig. 4b). At the same time, several genes encoding the rate-limiting enzymes of the glycolytic pathway from glucose to pyruvate were also found upregulated, including glucose kinase (*glk*), fructose 6-phosphate kinase (*pfkA*_2), and pyruvate kinase (*pykF*) (Fig. 4b). However, it is worth noting that after *ldh* and *pfl* were knocked out, their isoenzyme genes *ldh*1 and *pflA*_2 were significantly upregulated (Fig. 4b).

### Enhancement of stress tolerance using the response regulator DR1558

**Fig. 5:**
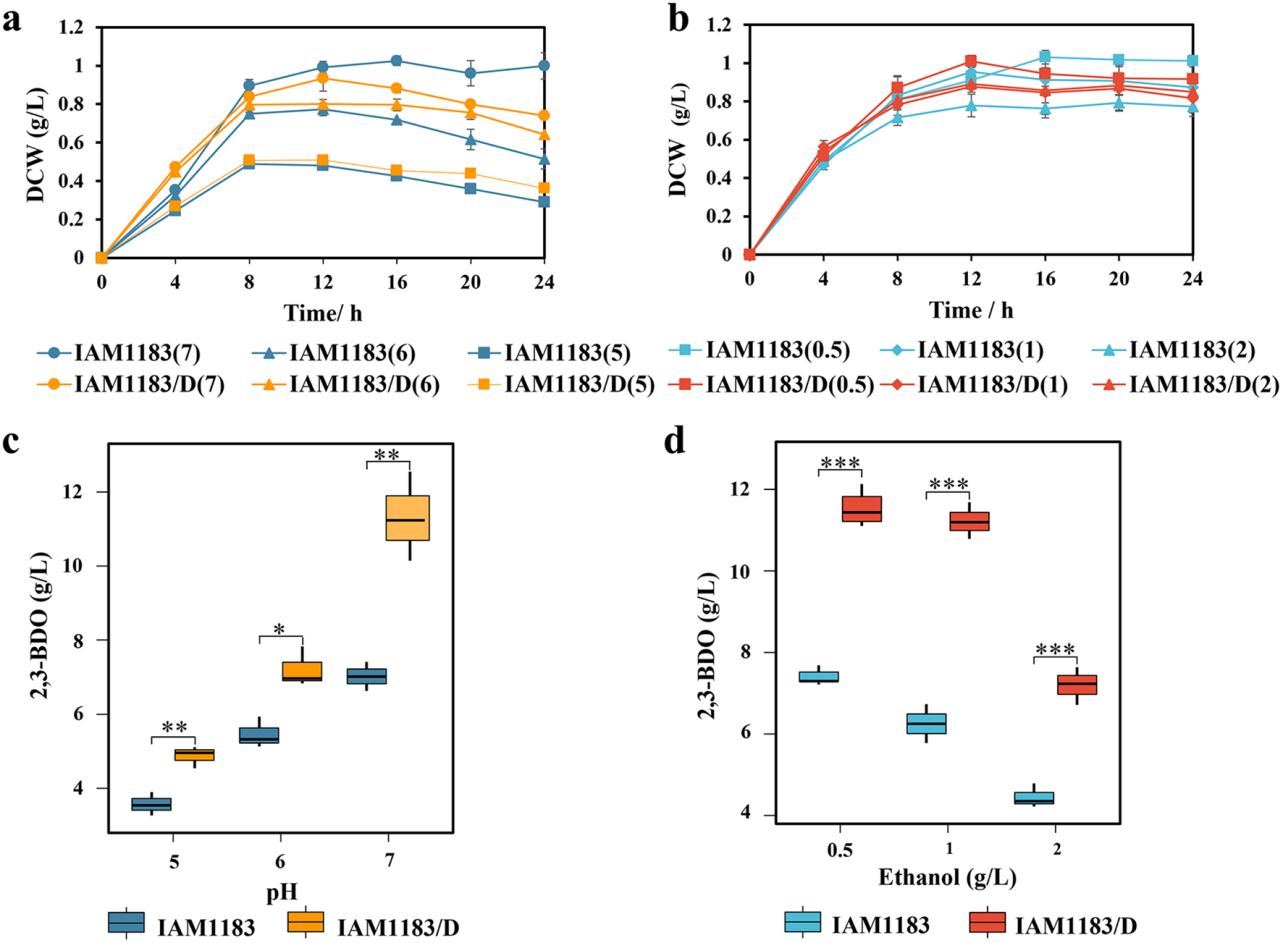
Effects of DR155 mutant on acid and ethanol tolerance and 2,3-butanediol production. **a** Growth curve of strains under different initial pH conditions of medium. **b** Growth curve of strains under different ethanol concentrations. **c** 2,3-BDO production at different pH values. **d** 2,3-BDO production at different ethanol concentrations. DCW means dry cell weight. *n* = 3 for each curve. Error bars show mean value ± SD. *P < 0.05, **P < 0.01, ***P < 0.001

The formation of 2,3-BDO is typically followed by a rapid pH decline brought on by the buildup of acidic by-products, and the accumulation of 2,3-BDO ceases once the pH falls below 5.0. Therefore, increasing stress resistance is a powerful strategy to increase the productivity of the strain*.* To evaluate the impact of DR1558 on biomass and 2,3-BDO production at different levels of acid and ethanol stress, IAM1183/D expressing DR1558 was grown in medium with different pH values and different ethanol concentrations for 24 h. The cultures of all the mutant strains had pH values between 5.0 and 6.8, while their ethanol titers were below 2 g/L during 24 h of cultivation. Thus, IAM1183/D was cultured at initial pH values of 7, 6, and 5 and ethanol concentrations of 0.5 g/L, 1 g/L, and 2 g/L to induce different degrees of acid or ethanol stress. The biomass and 2,3-BDO production of both the IAM1183 and IAM1183/D expressing DR1558 decreased along with the increase of acid and ethanol stress (Fig. 5). However, the stress-induced reduction of biomass in strain IAM1183/D was less pronounced than in IAM1183, indicating that DR1558 expression was conducive to improve the tolerance of *E. aerogene*s to acid and ethanol stress (Fig. 5a, b). Additionally, under varying levels of ethanol and acid stress, IAM1183/D expressing DR1558 produced much more 2,3-BDO than the wild type (Fig. 5c, d). In order to learn more about how the expression of DR1558 boosted the formation of 2,3-BDO, the mRNA expression of IAM1183/D was measured using qPCR (Fig. S2). The expression of major genes for the by-product production was downregulated while that of the 2,3-BDO production pathway genes was upregulated.

**Fig. 6:**
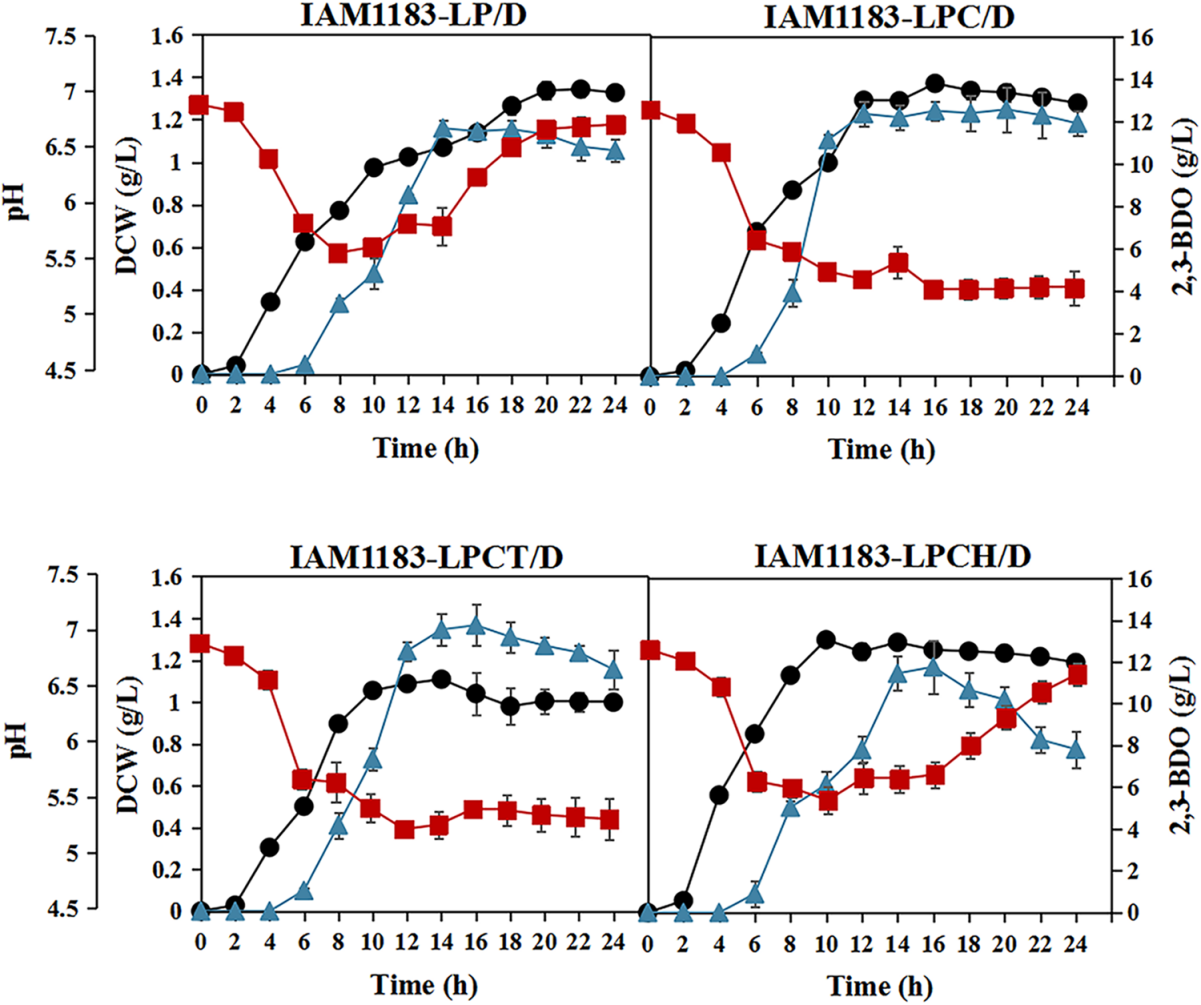
Effects of DR1558 on cell growth and 2,3-butanediol production in engineered *E. aerogenes.* Symbols: DCW (black-filled circle), 2,3-BDO (blue-filled triangle), and pH (red-filled square). DCW means dry cell weight. *n* = 3 for each curve. Error bars show mean value ± SD

To further improve strain proliferation and 2,3-BDO synthesis, the plasmid pET-28a-1558 was introduced into strains IAM1183-LP, IAM1183-LPC, IAM183-LPCT, and IAM183-LPCT. As expected, the biomass of all mutant strains was further increased (Fig. 6). Meanwhile, the heterologous expression of DR1558 significantly increased the productivity of 2,3-BDO and the substrate utilization in the mutant strains (Table 5), most notably in the engineered strains IAM1183-LPC/D and IAM1183-LPCT/D (Fig. 6). When the response regulator DR1558 was not introduced, strains IAM1183-LPC and IAM1183-LPCT reached 12.22 g/L and 13.33 g/L, respectively, after 24 h of shake flask fermentation (Table 4). However, when the response regulator DR1558 was introduced, strains IAM1183 LPC/D and IAM183 LPCT/D already reached 12.57 g/L and 13.76 g/L at 12 h and 16 h of fermentation, respectively, with 78% and 55% increase in productivity, respectively, compared to that without DR1558 expression. However, it is noteworthy that the 2,3-BDO titers of the mutant strains except mutant IAM1183LP/D did not increase or even decreased after 24 h of fermentation (Table 5), a phenomenon that may be attributed to the insufficiency of carbon source due to the accelerated depletion of the carbon source facilitated by the high biomass. Upon the carbon source depletion, 2,3-BDO acted as an alternate carbon source to maintain cell growth.

**Table 5:** Flask cultivation with strains expressing DR1558

Strains	Lactate (g/L)	Formate (g/L)	Acetate (g/L)	Succinate (g/L)	Pyruvate (g/L)	Ethanol (g/L)	Acetoin (g/L)	2,3-BDO (g/L)	Glucose (g/L)
IAM1183LP/D	0.15 ± 0.15	0.25 ± 0.02	1.05 ± 0.06	0.86 ± 0.04	0.18 ± 0.01	0.97 ± 0.08	1.87 ± 0.16	10.55 ± 0.83	2.35 ± 0.41
IAM1183-LPC/D	1.28 ± 0.07	0.63 ± 0.03	0.52 ± 0.07	0.48 ± 0.05	0.18 ± 0.01	0.89 ± 0.10	2.47 ± 0.06	11.92 ± 0.59	1.71 ± 0.15
IAM1183-LPCT/D	1.53 ± 0.05	0.41 ± 0.06	0.32 ± 0.05	0.65 ± 0.08	0.22 ± 0.01	0.68 ± 0.02	3.68 ± 0.05	11.56 ± 0.93	2.01 ± 0.28
IAM1183-LPCH/D	1.02 ± 0.03	0.27 ± 0.01	0.79 ± 0.02	0.27 ± 0.15	0.33 ± 0.02	0.43 ± 0.04	2.87 ± 0.13	7.82 ± 0.61	2.12 ± 0.50

### Optimization of the carbon source for engineered *E. aerogenes*

**Fig. 7:**
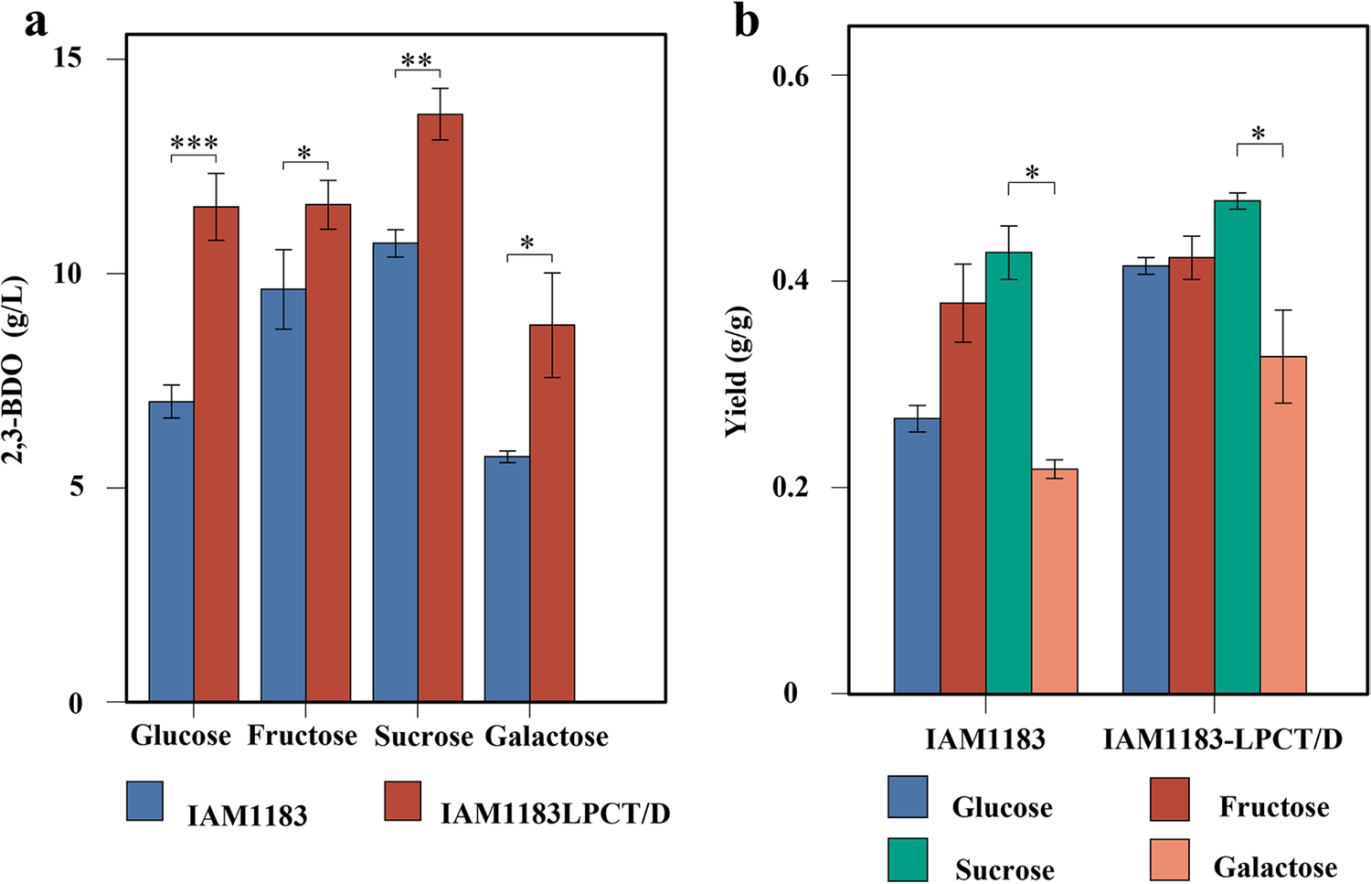
Comparison of 2,3-butanediol titer and yield of IAM1183-LPCT/D in different carbon sources. **a** Titer. **b** Yield. Error bars show mean value ± SD. *P < 0.05, **P < 0.01, ***P < 0.001

A suitable carbon source is crucial for biomass accumulation and microbial synthesis of target products (Oliviero et al. 2022; Gao et al. 2022). With this aim, the optimally engineered strain IAM1183-LPCT/D and the original strain IAM1183 were shake-flask fermented for 24 h with four different sugars to assess the 2,3-BDO titer and yield from each substrate (Fig. 7). The 2,3-BDO production of the IAM1183-LPCT/D was significantly increased compared with that of IAM1183 from all the four carbon sources. When glucose was used as the substrate, the titer of 2,3-BDO produced by IAM1183-LPCT/D increased most significantly compared to the original strain IAM1183, by 64.71%, and its glucose conversion rate increased from 0.27 g/g for IAM1183 to 0.41 g/g for IAM1183-LPCT/D. Moreover, IAM1183-LPCT/D showed the highest titer of 2,3-BDO (14.1 g/L) with sucrose as the carbon source, 20% higher than with glucose. It showed the lowest titer of 8.8 g/L from galactose. And the IAM1183-LPCT/D produced 2,3-BDO at the yield more than 0.4 g/g with glucose, fructose, and sucrose, among which the conversion rate under sucrose is the highest, up to 0.49 g/g (Fig. 7b).

### Batch fermentation in a 5-L fermenter

**Fig. 8:**
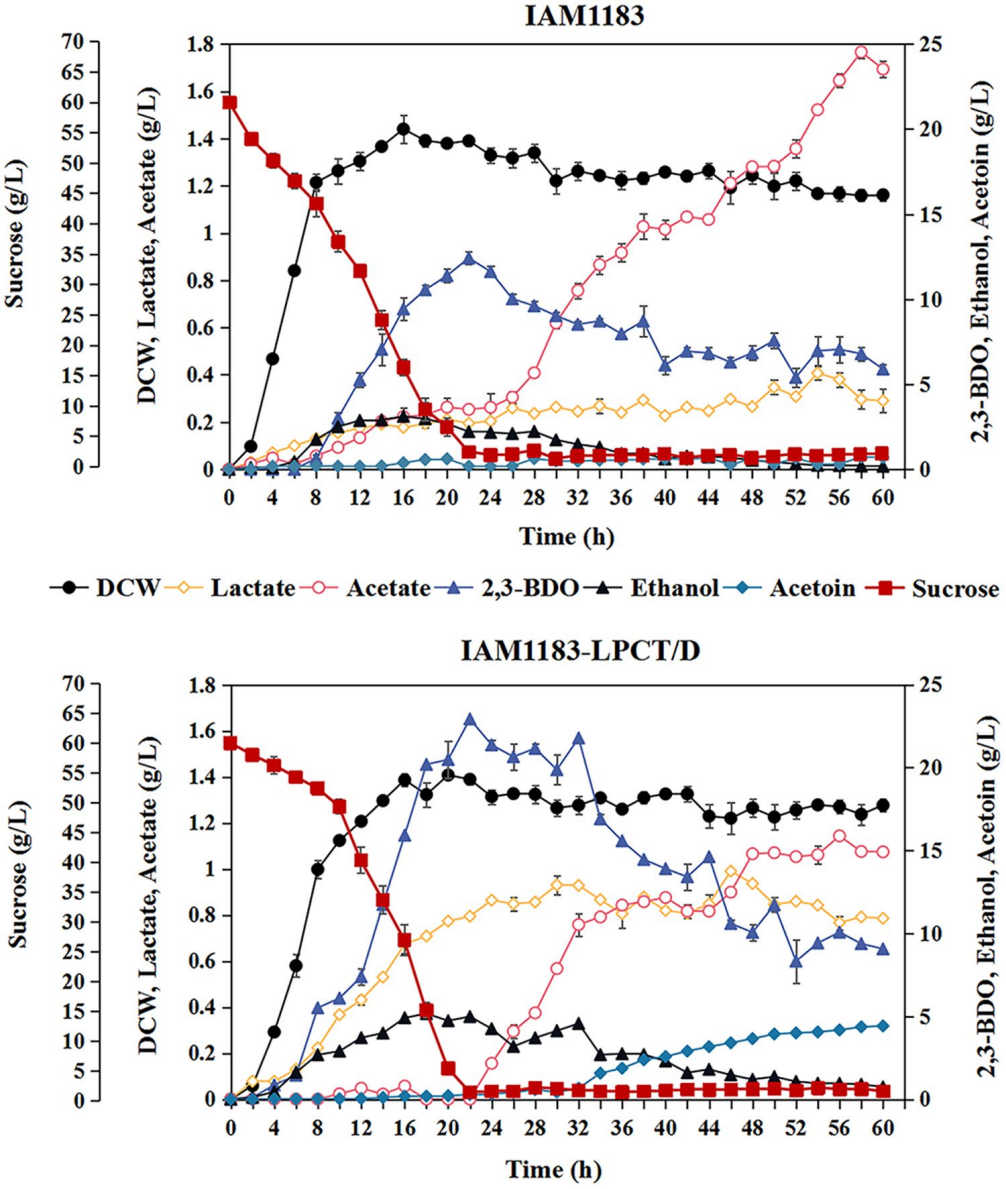
The fed-batch fermentation for 2,3-butanediol production in IAM1183 and IAM1183-LPCT/D. *n* = 2 for each curve. Error bars show mean value ± SD

After shake-flask fermentation, we attempted to investigate 2,3-BDO production by the IAM1183-LPCT/D compared to IAM1183. Figure 8 shows that the growth of both IAM1183 and IAM1183-LPCT/D reached a plateau after 16 h, with comparable biomass levels. At the same time, the 2,3-BDO titers of IAM1183 and IAM1183-LPCT/D decreased steadily with the extension of fermentation time after reaching the peak yield of 2,3-BDO after 22 h. The titer, yield, and productivity of 2,3-BDO of IAM1183-LPCT/D at 22 h were 22.93 g/L, 0.40 g/g, and 1.04 g/(L·h), respectively, which were 85%, 82%, and 86% higher compared to the original strain IAM1183 (Table 6). At 60 h, the titer of 2,3-BDO from IAM1183-LPCT/D was only 9.07 g/L, which was 60% lower than the titer of 2,3-BDO at 22 h (Table 6). It is worth noting that when the titer of 2,3-BDO reaches its peak at 22 h, sucrose in the medium was nearly exhausted (1.53 g/L), which may be a reason for the significant decline of the titer of 2,3-BDO in the later stage.

**Table 6:** Results of fermentation with IAM1183 and IAM1183-LPCT/D

Strains	22-h fermentation					
	Titer (g/L)	Yield (g/g)	Productivity (g/(L·h))	Titer (g/L)	Yield (g/g)	Productivity (g/(L·h))
IAM1183	12.42	0.22	0.56	5.89	0.10	0.10
IAM1183-LPCT/D	22.93	0.40	1.04	9.07	0.16	0.16

Regarding by-product production, formate (< 0.2 g/L) and succinate (< 0.1 g/L) were very low in both the original strain and the engineered strain (data not shown). However, after 22 h of fermentation, the by-product titers of acetate and acetoin gradually increased with the decrease of 2,3-BDO concentration, which may be caused by an imbalanced redox ratio inside the cells (Lee et al. 2021).

## Discussion

Metabolic engineering has evolved into a more systematic and higher throughput discipline, sometimes referred to as “systematic metabolic engineering” (Choi et al. 2019), which has been adopted in recent years to facilitate the production of high-performance strains (Li et al. 2023). In this work, to rationally obtain the desired phenotype, the effects of gene deletions related to by-product production on the growth and metabolic status of strains were determined using genetic engineering. Moreover, by using the combinatorial gene knockout strategy, the by-product titer decreased significantly while the titer of 2,3-BDO increased by 90%. Finally, by expressing a multi-stress tolerance response regulator, a dynamic tolerance engineering strategy was implemented to significantly improve the productivity of 2,3-BDO in *E. aerogenes.*

Under the guidance of the metabolic pathway model of *E. aerogenes* (Fig. 1), five by-products were identified to play an important role in inhibiting the production of 2,3-BDO in *E. aerogenes*. There are two major challenges that hinder the efficient production of 2,3-BDO: the low metabolic flux of the 2,3-BDO pathway and the inherent toxicity of compounds such as high alcohols and carboxylic acids (Lu et al. 2022; Park et al. 2020a, b). Meanwhile, trade-offs between biomass generation and target product production, redox balance, and intricate inhibitory control frequently make it difficult to optimize metabolic fluxes (Lee and Kim 2015). To address these issues, both static regulatory strategies, such as the use of genetic engineering to regulate product synthesis (Ge et al. 2020; Lu et al. 2022), precursor supply (Soma et al. 2022), and energy metabolism (Boecker et al. 2021; Jung et al. 2021), and dynamic regulatory strategies, such as expression component engineering (Wei et al. 2022), have been developed. Here, static and dynamic regulation strategies were combined to increase the production of 2,3-BDO. First, based on the metabolic pathway map of *E. aerogenes* and its transcriptome analysis, five key by-product genes (*ldh*, *pfl*, *ppc*, *pta*, *acdh*) were screened and used to construct different gene deletion mutant strains. Although four of these five targets have been reported to contribute to the production of 2,3-BDO (Guo et al. 2014; Thapa et al. 2019; Ge et al. 2020), most studies have compared potencies only at a certain timepoint. However, it is critical to study the changes in metabolites in real time, especially for 2,3-BDO production where negative feedback regulation is present. According to the time course production of 2,3-BDO, it is clear that the *ldh* deletion mutant strain has the highest productivity and the *ppc* deletion mutant strain has the lowest productivity. However, after 24 h of fermentation, the *pfl* deletion mutant strain had the highest production of 2,3-BDO, and the *ppc* deletion mutant strain had a higher production than the *ldh* deletion mutant strain, likely associated with the growth condition such as pH. It is well known that *ldh* deletion alleviates the acidification rate of the medium (Maina et al. 2022), thus promoting the growth amount of the strain and further accelerating the amount of substrate consumed. However, 2,3-BDO may act as a backup carbon source (Xiao and Xu 2007), and thus, the 2,3-BDO production of the *ldh*-deficient mutant strain gradually starts to decrease in the later stage of fermentation. Succinate is also an important by-product affecting the titer of 2,3-BDO. However, less research has been done in recent years to limit the succinate production (Thapa et al. 2019). Instead, most studies have focused on limiting lactate (Ge et al. 2020; Wu et al. 2021; Chu et al. 2021). Thapa et al. attempted to knock out the *mdh* gene encoding malate dehydrogenase to inhibit succinate production and further enhance 2,3-BDO production and then showed that 2,3-BDO was not significantly enhanced (Thapa et al. 2019). Among the metabolic pathways of succinate synthesis in microorganisms, the main pathway is the carboxylation of phosphoenolpyruvate to oxaloacetate, followed by the generation of succinate from oxaloacetate via the reduction branch of the tricarboxylic acid cycle (Zhang et al. 2017). The *ppc*-encoded phosphoenolpyruvate carboxylase is an important enzyme responsible for the carboxylation reaction of phosphoenolpyruvate, a compound reaction that converts phosphoenolpyruvate (C3 metabolite) into a centrally metabolized C4 metabolite (Zhang et al. 2017). To our knowledge, this is the first report on the effect of *ppc* gene deletion on 2,3-BDO production. This study showed that the deletion of *ppc* gene greatly inhibited the growth of the mutant strain, but its 2,3-BDO production reached 10.90 g/L after 24 h of fermentation, which was 55% higher compared to the wild type. This result suggests the importance of the phosphoenolpyruvate carboxylase encoded by the *ppc* gene to the cellular physiology of *E. aerogenes*.

In the present study, the metabolic flux of 2,3-BDO was further enhanced by reconfiguring the pyruvate metabolic network pathway to promote the production of 2,3-BDO. Given the importance of both biomass and 2,3-BDO titer, the double knockout mutant strain IAM1183-LP (*Δldh Δpfl*) was used as the starting strain (Lu et al. 2022), and three knockout mutant strains were constructed successively, among which the mutant strain IAM1183-LPCT (*Δldh Δpfl Δppc Δpta*) after 24 h of fermentation yielded 2,3-BDO at 13.33 g/L, which was 90% higher compared to the wild-type strain. Moreover, the multi-gene knockout did not affect the growth (Fig. 3). Thus, it is hypothesized that the promotion effect of *ldh* and *pta* deletion on the growth may aid in mitigating the growth inhibition caused by *pfl* and *ppc* deletion. In addition, the combined deletion of *ldh*, *pfl*, *ppc*, and *pta* genes significantly increased the expression of key enzyme genes in the glycolytic and 2,3-BDO synthesis pathways based on the results of transcriptome analysis, suggesting that limiting the production of by-products is an effective means to increase the metabolic flux of the synthesized target products.

A dynamic tolerance engineering strategy was designed by introducing the deinococcal response regulator DR1558 to improve microbial productivity and relieve metabolic burden. Engineering strategies to improve cellular stress resistance such as adaptive evolution (Huang et al. 2023; Zhou et al. 2023), regulatory factor introduction (Wu et al. 2021), tolerance target screening (Li et al. 2023; Cámara et al. 2022), and transport engineering (Mutanda et al. 2022) are now widely developed. Among them, the introduction of regulatory factors is a useful strategy to improve tolerance to target compounds even in the face of unknown toxicity mechanisms. To date, there are various stress threats during microbial fermentation, such as acid stress (Yao et al. 2022), ethanol stress (de Moura Ferreira et al. 2022), high osmotic stress (Liu et al. 2022), and high-temperature stress (Phong et al. 2022). *D. radiodurans* is one of the most stress-tolerant species and has been described as “the hardiest bacteria on Earth.” Many studies have demonstrated that the introduction of deinococcal response regulators, such as IrrE and DR1558, into the two-component system of a heterologous host can enhance stress tolerance, including radiation tolerance, osmotolerance, salt tolerance, alcohol tolerance, and acid tolerance (Guo et al. 2017; Park et al. 2019; Park et al. 2020a, b). Among them, the DR1558 response regulator is the most functionally rich. In the present study, the introduction of DR1558 allowed to significantly increase biomass accumulation and metabolic production of 2,3-BDO by the engineered strain against acid and ethanol stresses. The qPCR analysis was performed to further determine the effect of DR1558 expression on the gene expression of host strains. The results showed that the introduction of DR1558 mostly downregulated genes for by-product biosynthesis competing with 2,3-BDO and cofactor NADH, while the gene expressions of key enzymes in the synthesis pathway of 2,3-BDO were significantly upregulated. This phenomenon is consistent with the results of transcriptome analysis by Park et al. (2020a, b), which further indicates that the expression of DR1558 has a wide range of regulatory effects on cell metabolism. As expected, the biomass accumulation of all combined knockout mutants was significantly improved with the introduction of DR1558. At the same time, the 2,3-BDO productivity of the mutants was all improved, with the mutant IAM1183-LPC having the highest magnitude with a 78% increase. This suggests that the introduction of DR1558 into a heterologous host is an effective means to improve the production performance of the host strain 2,3-BDO.

Besides strain engineering, optimization of carbon sources is an important strategy to improve the yield of target products synthesized by microorganisms and the efficiency of substrate conversion (Oliviero et al. 2022; Gao et al. 2022). Currently, the main carbon sources used in the fermentation production of 2,3-BDO are glucose, fructose, sucrose, lactose, xylose, and inulin. Competition in raw materials has always been fundamental between the production of 2,3-BDO by fermentation and chemical synthesis, since a cheap and abundant source of carbon is one of the most important prerequisites for the development of industrial production (Um et al. 2017; Lee and Seo 2019; Kim et al. 2020). In this study, the yield of 2,3-BDO from glucose, fructose, and sucrose of mutant IAM1183-LPCT/D was all above 0.4 g/g, among which sucrose had the highest yield of 0.49 g/g. Sucrose has always been one of the ideal low-cost raw materials for industrial raw materials. At the same time, glucose and fructose are currently the two most common monosaccharides. Their low-cost commercial feedstocks are often used in industrial fermentation, such as raw sugar, cornstarch hydrolysates, and sugarcane molasses (Jung et al. 2015). Thus, the high conversion rate of 2,3-BDO produced by the engineered bacterium IAM1183-LPCT/D in the presence of glucose, fructose, and sucrose will facilitate the use of low-cost feedstocks in industrial fermentation and thus boost the competitiveness of 2,3-BDO production via biotransformation.

In recent years, with the rapid development of the fields of genetic engineering, evolutionary engineering, and omics, the tools and strategies of metabolic engineering have been further broadened, enabling increasingly systematic engineering to achieve superior performance of microbial strains (Park et al. 2014; Yang et al. 2020; Li et al. 2023). So far, there have been many studies on the production of 2,3-BDO using *Klebsiella* sp., *Bacillus* sp., and *Saccharomyces* sp. in which the highest titer has reached up to 100 g/L (Ma et al. 2009; Kim et al. 2012). Although the titer in some studies is higher than this study, 2,3-BDO is accompanied with significant amount of by-products, which creates technical difficulties and increases the cost for subsequent isolation and purification. Moreover, in those studies, high-concentration and high-purity carbon sources have been fed to the engineered strains to achieve a higher titer of target products, which undoubtedly increases the cost of raw materials. The strategy chosen in this study allowed the engineered strains to obtain a significant increase in production (titer, yield, and productivity) while at the same time showing a significant decrease in by-product production, which may significantly reduce the cost for fermentation and subsequent isolation and purification. Fermentation production of 2,3-BDO in the engineered strains could be further improved by optimizing the fermentation parameters such as aeration, agitation, pH, temperature, inoculum amount, and substrate concentration (Song et al. 2019).

In summary, a high-level and low-cost 2,3-BDO production platform based on *E. aerogenes* was established based on a systematic metabolic engineering strategy, consisting of reducing the accumulation of by-products, improving the tolerance of host cells to toxic products, and optimizing the conditions of fractionated fermentation. The engineered *E. aerogenes* strain IAM1183-LPCT/D produced 22.93 g/L of 2,3-BDO in 5 L fermentation with a yield of 0.40 g/g and a productivity of 1.04 g/(L·h), which was 85%, 82%, and 86% higher than the original strain IAM1183, respectively. The by-products in the fermentation broth are mainly ethanol and lactate. Our results show the potential of systematic metabolic engineering strategies to improve the efficiency of microbial cell factories, while also providing valuable insights for using other strains to produce different target products from renewable and cost-effective carbon sources.

## Supplementary Information

Below is the link to the electronic supplementary material.

Supplementary file1 (PDF 291 KB)

## Data Availability

The data that support the figures within this paper and other findings of this study are available from the corresponding author upon reasonable request.
