# Supplementary material for: Systemic metabolic engineering of Enterobacter aerogenes for efficient 2,3-butanediol production
Source: Appl Microbiol Biotechnol. 2024 Jan 19;108(1):146. doi: 10.1007/s00253-023-12911-8 (PMC10798932; doi:10.1007/s00253-023-12911-8)
Supplement: Supplementary file 1 — Supplementary file1 (PDF 291 KB) [file 253_2023_12911_MOESM1_ESM.pdf]

Applied Microbiology and Biotechnology

Systemic metabolic engineering of *Enterobacter aerogenes* for efficient 2,3-butanediol production

Ping Lu[1], Ruoxuan Bai[1], Ting Gao[1], Jiale Chen[1], Ke Jiang[1], Yalun Zhu[1], Ye Lu[1], Shuting Zhang[1], Fangxu Xu[2] and Hongxin Zhao[1]*

[1]Zhejiang Province Key Laboratory of Plant Secondary Metabolism and Regulation, College of Life Sciences and Medicine, Zhejiang Sci-Tech University, Hangzhou 310018, China

[2]Liaoning Province Key Laboratory of Cordyceps Militaris with Functional Value, Experimental Teaching Center, Shenyang Normal University, Shenyang 110034, China

Correspondence should be addressed to Hongxin Zhao, Zhejiang Province Key Laboratory of Plant Secondary Metabolism and Regulation, College of Life Sciences and Medicine, Zhejiang Sci-Tech University, Hangzhou 310018, China. Tel: +86-571-86843195; E-mail: bxxbj2003@gmail.com

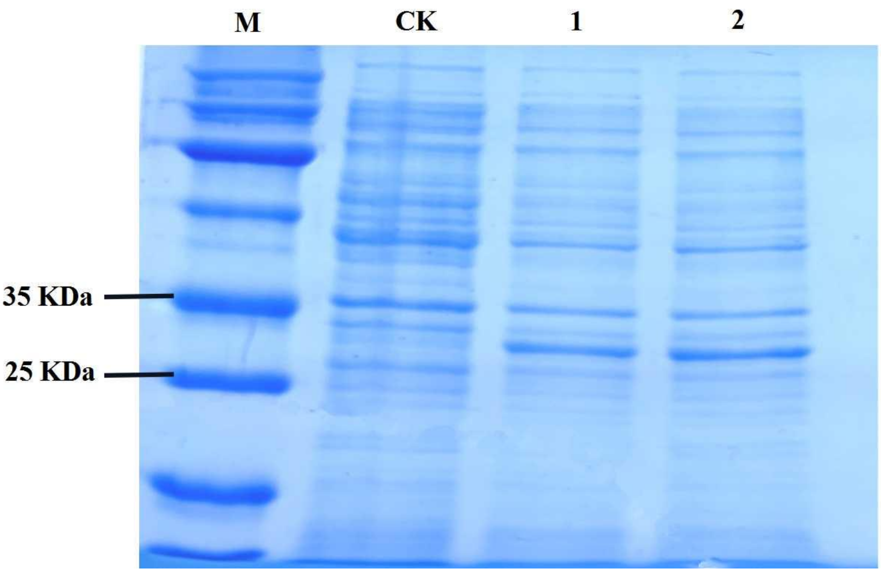

Fig. S1 DR1558 expression detection on SDS-PAGE. Note: First lane: Protein Marker; CK: IAM1183; 1: IAM1183/D; 2: IAM1183/D.

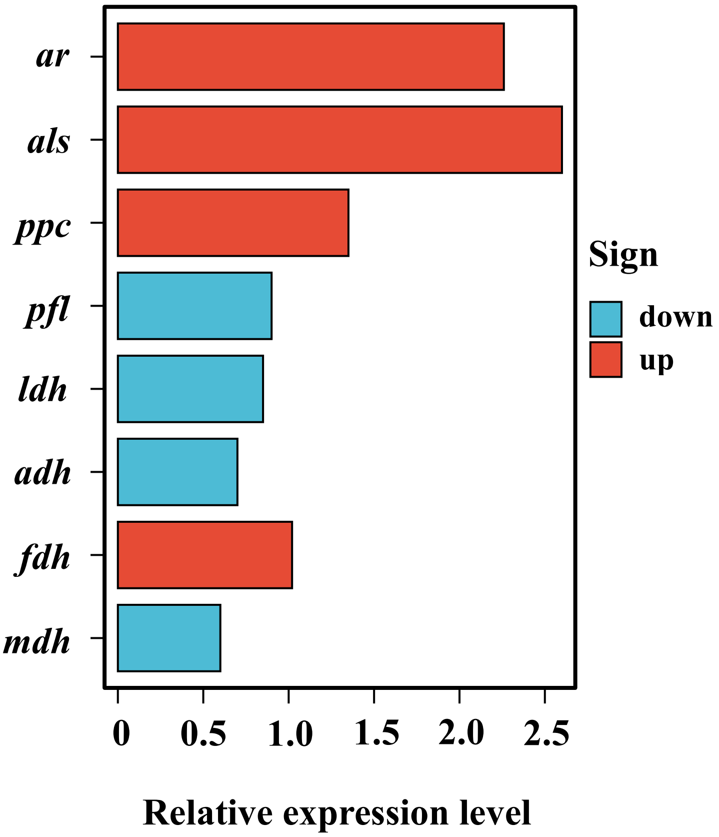

Fig. S2 Relative expression levels of *ar, als, ppc, pfl, ldh, adh, fdh and mdh* analyzed by RT-qPCR in strain IAM1183/D. Note: the set expression level of each gene of the IAM1183 was 1.

Table S1 Effective genes and functions of transcriptional upregulation in the IAM1183-LPCT pyruvate metabolic pathway

Gene_id	Gene name	Gene description
EAChromosome_1_04283	*glk*	Glucokinase
EAChromosome_1_00741	*pgi*	Glucose-6-phosphate isomerase
EAChromosome_1_00552	*pfkA_2*	6-phosphofructokinase isozyme 1
EAChromosome_1_04850	*pgk*	Phosphoglycerate kinase
EAChromosome_1_04649	*eno*	Enolase
EAChromosome_1_01994	*gpmA*	3-bisphosphoglycerate-dependent phosphoglycerate mutase
EAChromosome_1_03780	*pykA*	Pyruvate kinase II
EAChromosome_1_03507	*pykF*	Pyruvate kinase I
EAChromosome_1_01293	*aceF*	Dihydrolipoyllysine-residue acetyltransferase component of pyruvate dehydrogenase complex
EAChromosome_1_01691	*gltA_1*	Citrate synthase
EAChromosome_1_03192	*fumA_1*	Fumarate hydratase class I
EAChromosome_1_02423	*icd*	Isocitrate dehydrogenase [NADP]
EAChromosome_1_05231	*accC_2*	Biotin carboxylase
EAChromosome_1_01383	*accA*	Acetyl-coenzyme A carboxylase carboxyl transferase subunit alpha
EAChromosome_1_03053	*ldh_1*	L-lactate dehydrogenase 1
EAChromosome_1_02337	*ghrA_1*	Glyoxylate/hydroxypyruvate reductase A
EAChromosome_1_02644	*adhC2*	NADP-dependent alcohol dehydrogenase
EAChromosome_1_02139	*poxB*	Pyruvate dehydrogenase [ubiquinone]
EAChromosome_1_03050	*sad*	Succinate semialdehyde dehydrogenase [$NAD(P)^+$] Sad
EAChromosome_1_02166	*pflA_2*	Pyruvate formate-lyase 1
EAChromosome_1_00598	*ilvA*	L-threonine dehydratase
EAChromosome_1_00415	*ilvB_1*	Acetolactate synthase isozyme
EAChromosome_1_00600	*ilvC*	Ketol-acid reductoisomerase
EAChromosome_1_00597	*ilvD_1*	Dihydroxy-acid dehydratase

EAChromosome_1_00594	*ilvG*	Acetolactate synthase isozyme
EAChromosome_1_00596	*ilvE*	Branched-chain-amino-acid aminotransferase
EAChromosome_1_01254	*ilvH*	Acetolactate synthase isozyme
EAChromosome_1_01253	*ilvI*	Acetolactate synthase isozyme
EAChromosome_1_00595	*ilvM*	Acetolactate synthase isozyme
EAChromosome_1_00414	*ilvN*	Acetolactate synthase isozyme
EAChromosome_1_03430	*alsD*	Alpha-acetolactate decarboxylase
EAChromosome_1_03432	*budC_2*	Diacetyl reductase [(S)-acetoin forming]
EAChromosome_1_03431	*budB*	Acetolactate synthase
